# The future of integrative neuroscience: The big questions

**DOI:** 10.3389/fnint.2023.1113238

**Published:** 2023-02-23

**Authors:** Catalin V. Buhusi, Sorinel A. Oprisan, Mona Buhusi

**Affiliations:** ^1^Interdisciplinary Program in Neuroscience, Department of Psychology, USTAR BioInnovations Center, Utah State University, Logan, UT, United States; ^2^Department of Physics and Astronomy, College of Charleston, Charleston, SC, United States

**Keywords:** neuroscience, integrative, memory, engram, training

## 1. Introduction

Neuroscience is ready to transcend the reductionist approach (Joyce and Shergill, 2018; Pessoa, 2022). The revolutionary integrative approach to synthesizing information from single neurons, circuits, and whole brain imaging and manipulations using methods derived from multiple disciplines: chemistry, genetics, molecular and cellular biology, systems biology, and behavioral sciences, is producing “big data” sets. Here we explore several “big questions” posed by the integrative approach: How to integrate heterogeneous neuroscience information? How to train the workforce for this approach? What resources are needed for this integrative revolution? And also: Who stands to benefit?

Take studying the neural bases of memory: Memories of past experiences are the essence of who we are, and are key to our awareness of time in our everyday lives. When no new memories are formed, people are condemned to an eternal present. Retrograde amnesia prevents recalling memories before the event that caused it. Occasionally, some experience false memories of events that never happened. To distinguish *memory* from *expectation* and *perception*, Plato and Aristotle proposed that events that have already happened leave in our mind “*memory traces”* (De Brigard, 2014). To describe the physical representation of putative *memory traces*, Semon introduced the term “*engram*” (Semon, 1904). Several key events in *disparate fields* permitted the study of engrams: Watson and Crick's discovery of DNA, Donald Hebb's theory that “neurons that fire together wire together,” Milner's studies of amnesic patient H.M., and Lømo's discovery of long-term potentiation (Asok et al., 2019). Progress culminated in recent years with the development of integrative *engram technologies* capable of identifying, visualizing, and manipulating engram formation, storage and recall.

## 2. An integrative approach to memory

### 2.1. Engram technologies

Engram technologies integrate methods from multiple fields: *transgenic/genetic manipulation*, permitting expression of neuronal markers to label engrams (Navabpour et al., 2020); *optogenetics*, allowing activation (or silencing) of engram cells using light (Kim et al., 2017); *chemogenetics*, allowing designer drug activation / inhibition of neurons (Roth, 2016); *electrophysiology*, allowing recording of neuronal electrical activity in behaving animals in real time (Chorev et al., 2009); *modern microscopy*, allowing the visualization of engram cells in real time, e.g., using miniaturized microscopes (Carrillo-Reid et al., 2017; Yang and Yuste, 2017); and *behavioral techniques*, allowing the dissection of engram formation, consolidation, recall, and update (reviewed in Ortega-de San Luis and Ryan, 2022).

Using this integrative approach, scientists were recently able to create and manipulate (false) memories (Lau et al., 2020). Mice were exposed to a conditioned stimulus (CS) terminating in footshock under drugs that allowed labeling the neurons recruited to the engram of this experience. Visualization of the “CS-footshock” engram indicated that engram cells remain highly excitable for several hours after conditioning. While the engram was still excitable, a second, neutral stimulus was presented: A new engram, partially overlapping with the “CS-footshock” engram, developed. Next day, mice froze to the neutral stimulus, showing that they (falsely) recalled being shocked in the presence of the neutral stimulus. False memories were prevented by optogenetically silencing the “CS-footshock” engram, or by presenting the neutral stimulus 24 h after conditioning, suggesting a temporal window during which engrams can be associated (Lau et al., 2020). Such groundbreaking manipulations could not have been possible without integration of multiple methods from varied sciences in the same study.

### 2.2. Why “integrative” neuroscience?

**Figure 1:**
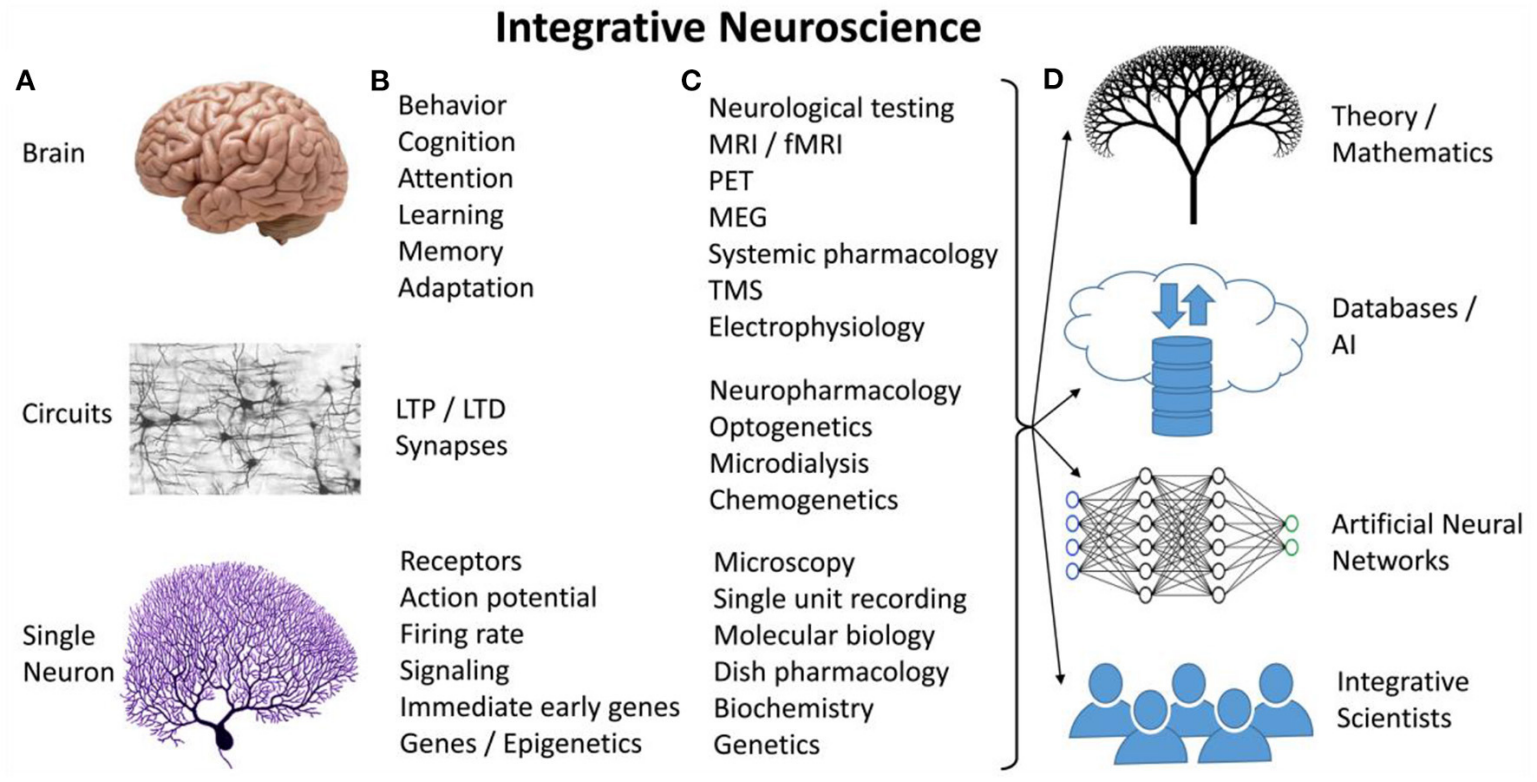
Integrative neuroscience encompasses multiple levels of analyses **(A)**, and integrates “big data” **(B)** collected using diverse methods **(C)** to uncover relationships and phenomena that transcend levels. **(D)** Several solutions to the integration problem.

Neuroscience is currently investigating multiple *levels of analysis*—from single neuron, to circuits, to whole brain explorations (Figure 1A)—and a multitude of *mechanisms*—genes, receptors, electrical activity, circuit activation and inhibition, and behavior in animals and people with or without specific diseased conditions (Figure 1B)—using a multitude of *methods* derived from varied sciences—chemistry, physics, genetics, molecular and cellular biology, behavior, and computer science (Figure 1C). Two issues are paramount: First, the quantity of data collected is staggering. Second, scientists are sequestered in their “local field,” bound by methods used, language, and interest. Thus, the need for a paradigm shift toward an “Integrative Neuroscience” (INS) approach that transcends the levels of analysis and allows the discovery of new properties or natural laws that apply at the integrative level (Grillner et al., 2005) (Figure 1D). Several “big questions” are outstanding:

## 3. The big questions

### 3.1. How to integrate heterogeneous neuroscience information?

Several answers are provided in Figure 1D; they all have strengths and weaknesses. First, integration can occur at theoretical level, using Cybernetics, a mathematical theory of how systems work (Figure 1D top). Cybernetics' strength is its formalism and its well-studied notions, immediately applicable to neuroscience: communication, transformation of information, feedback, and stability. Such theories are under development (e.g., Chauvet, 2005), with the hope that soon they will integrate real data.

A more practical approach is to store raw data into databases, either local or “in the cloud” (Gordon, 2003) (Figure 1D second from top). One drawback is the level of detail optimal for integration, similar to seeing the forest for the trees: Less detail helps integration but decreases accuracy; too much detail provides accuracy but prevents integration. Moreover, storing data in databases helps identifying correlations between phenomena at different levels, but new theories or new levels of understanding are not expected to simply emerge. To search, understand, and integrate “big data” one may need to use artificial intelligence, a combination of artificial neural networks (ANNs), fuzzy systems, and decision algorithms, to develop new representations.

ANNs have been used for more than half a century to understand INS data (McCulloch and Pitts, 1943) (Figure 1D second from bottom). As scientists that have used ANNs and fuzzy systems to model neuroscience phenomena the authors can attest to their power (Buhusi et al., 1998; Buhusi, 2000), as to their occasional ineffectiveness (e.g., Buhusi et al., 2016, 2018): ANNs develop new representational spaces in their “deep layers” where the solution is “hidden”; decoding it—let alone understanding it—in human terms is often very difficult if not impossible (Lamoureux et al., 1998). To put it simply, should ANNs be large enough—using many nodes—and “deep” enough—using many layers—they are likely to provide representations / solutions as complex as the brain itself, requiring the same level of effort for its understanding as the original data set. To use ANNs solutions, a person (or team) is needed to extract and interpret it. Which brings one to the simplest—yet the most difficult—solution to the integration problem: Training an INS workforce (Figure 1D bottom).

### 3.2. Who will make the integration?

The current system of scientific inquiry is based on ever higher levels of specialization. This results in the sub-fields of neuroscience being rather “self-sufficient” and “isolated” from other levels. Only in the last decades have truly multi-disciplinary approaches emerged from isolated laboratories. Training in these laboratories provides “integrative” development for a limited number of select scientists, too few to determine a change of the entire field. New “experts in one method” are constantly produced while the number of faculty positions lags well behind, thus providing little incentive for poly-specialization (Akil et al., 2016).

Yet, recognizing the need for INS training, a handful of PhD programs around the world have started adding “integrative” to their designation, aiming at training future neuroscientists in multiple methods, and more importantly, in the skills that would allow them to work efficiently in a team analyzing the same problem at multiple levels. University of Chicago, Duke University, Massachusetts Institute of Technology, University of Nevada, Stony Brook University, and University of Cardiff are among the universities offering INS training. The neuroscience curriculum of these institutions has been restructured to include courses on “Integrative Neuroscience” and on data analysis and computational modeling (e.g., “Bioinformatics,” and “Artificial Neural Networks”), and to also include student rotations through labs using various methods.

This is an excellent first step. Yet, not only more programs are needed to provide INS training to future PhDs, but “integrative training” should start before—possibly way before—PhD training. A bold example is provided by the new Integrative Science Department at Claremont Mckenna College (2022), dedicated to training future integrative scientists. Moreover, developing computer skills and a keen eye for looking at a situation from both a close- and far distance, are skills that can be taught earlier than college or PhD (Dube et al., 2017).

### 3.3. Who stands to benefit from the integrative revolution in neuroscience?

Given that neuroscience has already entered its era of technological applications (Altimus et al., 2020), not only academia, but industry, government—e.g., governmental funding agencies—as well as foundations, and ultimately, patients and the general population stand to benefit from the INS revolution. The National Institutes of Health are already funding neuroscience technology development through its BRAIN initiative (Jorgenson et al., 2015), and together with the National Science Foundation, are offering funding for multidisciplinary collaborative research (Plimpton, 2020) that brings innovations into the marketplace (Bates and Garbarini, 2014). Yet, considerably more funding is needed to transform out-of-date “one-method” labs into modern “integrative” labs.

While traditionally new methods are brought about by new generations of scientists, one should recognize that major changes (read “discoveries”) are now happening faster than within one generation, such that the workforce needs periodic re-training to take advantage of the explosion in new methods and technologies. As usual, academia trails behind industry, which is already used to transformative changes happening faster than ever, and manages to profit from the use of these new discoveries (Hyman, 2016). The general population already benefits from translating the INS revolution into the marketplace: self-driving vehicles, intelligent voice-interactive devices, and artificial intelligence software for various functions.

### 3.4. Will integrative engram technologies provide new treatments?

Patients—e.g., Alzheimer's patients—stand to benefit from seeing molecular and cellular targets rapidly evaluated at multiple levels of analysis, hopefully with better and faster treatment development. For example, integrative engram technologies recently revealed the molecular control of the temporal window at which engrams can be linked. CCR5, a C–C chemokine receptor expressed in hippocampal neurons and glia (Shen et al., 2022), is affected in conditions in which memory linking is either reduced (e.g., aging and Alzheimer's Disease) or enhanced (e.g., PTSD), and may also contribute to neuroinflammation (Necula et al., 2021). Age-related memory deficits are reversed by a CCR5 antagonist already approved by the FDA, a possible game-changer in cognitive decline therapy. Further integrative research regarding neuronal-, astrocytic- and microglial-interactions will pave the way for new therapeutic approaches in cognitive aging and neurodegenerative disorders.

## 4. Into the future

Due to the vast number of inter-connections between data and mechanisms at different levels of neuroscience analysis, integration is expected to create synergies: Pending a major restructuring of neuroscience training, future INS scientists will learn to think and work “integrative,” using multiple methods to investigate the same problem at multiple levels, to bring new problems, new applications, and new treatments within reach. This will benefit the general population and patients alike, such as those with memory impairments. Indeed, visualizing engrams is already possible in humans (Willems and Henke, 2021). In the future, engrams could be investigated in naturalistic situations—such as participants having dinner—by simultaneously recording their brain activity, location, gaze, body function indices like heart rate and blood pressure, what is said, and each participant's reaction (Kanter et al., 2022). Also, one will be able to trace the timing of molecular activity during memory development, storage, retrieval and updating in the whole brain. Integration will allow the identification of biomarkers to illuminate disease-related changes at multiple levels, from molecules, to cells, to whole brain, allowing for rapid steps toward understanding a disease and designing a treatment or prevention strategy. Finally, people with cognitive decline will be able to use soon-to-be ubiquitous non-invasive brain stimulation devices in their own homes, to support or enhance their memory function (Antal et al., 2022). One thing is sure: Neuroscience is an exciting field in the midst of an “integrative” revolution, with implications extending beyond basic, reductionist experiments, in directions that are stretching the limits of our imagination.

## Author contributions

CB and MB wrote the first draft of the manuscript. All authors contributed to manuscript revision, read, and approved the submitted version.
